# Draft Genome Sequence of Methicillin-Resistant *Staphylococcus aureus* CUHK_188 (ST188), a Health Care-Associated Bacteremic Isolate from Hong Kong

**DOI:** 10.1128/genomeA.00255-14

**Published:** 2014-04-17

**Authors:** Margaret Ip, Zheng Wang, Wai Yip Lam, Haokui Zhou, Stephen Tsui

**Affiliations:** a Department of Microbiology, the Chinese University of Hong Kong, the Prince of Wales Hospital, Hong Kong, China; b School of Biomedical Sciences, the Chinese University of Hong Kong, Shatin, Hong Kong, China

## Abstract

We report the draft genome sequence of a methicillin-resistant *Staphylococcus aureus* strain designated CUHK_188, isolated from a bacteremic patient undergoing treatment at a university teaching hospital in Hong Kong. This strain belongs to sequence type 188 (ST188), with *spa* type t189 and staphylococcal cassette chromosome *mec* type V.

## GENOME ANNOUNCEMENT

*Staphylococcus aureus* sequence type 188 (ST188) is a double-locus variant (DLV) of ST1, which includes MW2/USA400, the highly virulent and first known Pantón-Valentine leukocidin (PVL)-positive methicillin-resistant *S. aureus* (MRSA) strain (1). Methicillin resistance in ST188 has been increasingly described, particularly across the Asia-Pacific region. It has been reported in Hong Kong (2), many provinces of mainland China (3, 4), Korea (5), Malaysia (6), and Taiwan (7). Sporadic cases of community-associated MRSA infection have also been reported in Australia (8). The sequenced strain reported here, CUHK_188, is a health care-associated MRSA strain isolated from a 78-year-old female patient with MRSA bacteremia in a university teaching hospital in 2007. It belongs to *spa* type t189 and staphylococcal cassette chromosome *mec* type V (SCC*mec* V), and it is PVL negative. The multilocus sequence typing (MLST) seven-housekeeping-gene allelic pattern is 3-1-1-8-1-1-1 (ST188), a DLV of ST1 (http://saureus.mlst.net/). However, initial DNA microarray-based typing indicated large differences between ST188 and ST1 (2), suggesting the complex evolutionary processes of the ST188 clone.

We sequenced the CUHK_188 genome using the Ion Torrent platform (Ion PGM Sequencer, Torrent Suite software). A total of 1,236,897,130 bp in 6,438,819 reads was obtained. After quality control performed with PrinSeq (9), the sequence reads were *de novo* assembled using the Roche GS Assembler software. The CUHK_188 genome was assembled into 41 large contigs (*N*_50_, 153,524 bp; largest contig size, 271,458 bp; average length, 68,487 bp). The genome is approximately 2.81 Mb in length, with an average G+C content of 32.7%. The average coverage depth was 440×.

The genome was annotated using NMPDR RAST (10) and Geneious (Biomatters Ltd., New Zealand). A total of 2,700 coding DNA sequences (CDSs), 56 tRNA-coding genes, and 4 rRNA loci were detected, with 53% of the genes assigned to specific subsystem categories by RAST (10). Initial comparative analyses with the ST1 reference genome *S. aureus* MW2 (11) (accession no. NC_003923) highlighted a number of indels and mobile genetic element (MGE) differences. Of note, the prophage φSa2, which is present in MW2 and harbors the *lukSF* genes encoding PVL, is absent in CUHK_188. PHAST analysis (12) identified that the genome carries two intact prophage regions and one incomplete prophage region. The first intact prophage region of CUHK_188 shows high similarity with prophage φNM1 and harbors three virulence genes, i.e., homologues of the SAV0866, SAV1978, and SAV0862 genes (13). The second prophage shows high similarity with prophage φNM3 (13) and contains genes that encode modulators of the innate immune responses: staphylokinase (*sak*), chemotaxis inhibitory protein (*chp*), and staphylococcal complement inhibitor (*scn*). Both of these prophages were described in *S. aureus* strain Newman, which has been shown to play important roles in the pathogenesis of staphylococcal infections (14). The *sasX* gene, which is linked to the φSPβ-like prophage and enhanced MRSA nasal colonization in the Asian ST239 epidemic clone (15), was absent.

Further studies are under way with the CUHK_188 genome that will advance our understanding of the evolution of this emerging clone in the Asia-Pacific region.

### Nucleotide sequence accession numbers.

This whole-genome shotgun project has been deposited at DDBJ/EMBL/GenBank under the accession no. JFFV00000000. The version described in this paper is version JFFV01000000.

## References

[1] Centers for Disease Control and Prevention 1999 Four pediatric deaths from community-acquired methicillin-resistant *Staphylococcus aureus*—Minnesota and North Dakota, 1997–1999. JAMA 282:1123–1125. 10.1001/jama.282.12.1123-JWR0922-2-1 10501104

[2] Monecke S Coombs G Shore AC Coleman DC Akpaka P Borg M Chow H Ip M Jatzwauk L Jonas D Kadlec K Kearns A Laurent F O’Brien FG Pearson J Ruppelt A Schwarz S Scicluna E Slickers P Tan HL Weber S Ehricht R 2011 A field guide to pandemic, epidemic and sporadic clones of methicillin-resistant *Staphylococcus aureus*. PLoS One 6:e17936. 10.1371/journal.pone.0017936 21494333 PMC3071808

[3] Yu F Li T Huang X Xie J Xu Y Tu J Qin Z Parsons C Wang J Hu L Wang L 2012 Virulence gene profiling and molecular characterization of hospital-acquired *Staphylococcus aureus* isolates associated with bloodstream infection. Diagn. Microbiol. Infect. Dis. 74:363–368. 10.1016/j.diagmicrobio.2012.08.015 23021064

[4] He W Chen H Zhao C Zhang F Li H Wang Q Wang X Wang H 2013 Population structure and characterisation of *Staphylococcus aureus* from bacteraemia at multiple hospitals in China: association between antimicrobial resistance, toxin genes and genotypes. Int. J. Antimicrob. Agents 42:211–219. 10.1016/j.ijantimicag.2013.04.031 23871455

[5] Peck KR Baek JY Song JH Ko KS 2009 Comparison of genotypes and enterotoxin genes between Staphylococcus aureus isolates from blood and nasal colonizers in a Korean hospital. J. Korean Med. Sci. 24:585–591. 10.3346/jkms.2009.24.4.585 19654937 PMC2719184

[6] Ghaznavi-Rad E Nor Shamsudin M Sekawi Z Khoon LY Aziz MN Hamat RA Othman N Chong PP van Belkum A Ghasemzadeh-Moghaddam H Neela V 2010 Predominance and emergence of clones of hospital-acquired methicillin-resistant *Staphylococcus aureus* in Malaysia. J. Clin. Microbiol. 48:867–872. 10.1128/JCM.01112-09 20089756 PMC2832418

[7] Chen FJ Siu LK Lin JC Wang CH Lu PL 2012 Molecular typing and characterization of nasal carriage and community-onset infection methicillin-susceptible *Staphylococcus aureus* isolates in two Taiwan medical centers. BMC Infect. Dis. 12:343. 10.1186/1471-2334-12-343 23228040 PMC3522061

[8] Nimmo GR Coombs GW 2008 Community-associated methicillin-resistant *Staphylococcus aureus* (MRSA) in Australia. Int. J. Antimicrob. Agents 31:401–410. 10.1016/j.ijantimicag.2007.08.011 18342492

[9] Schmieder R Edwards R 2011 Quality control and preprocessing of metagenomic datasets. Bioinformatics 27:863–864. 10.1093/bioinformatics/btr026 21278185 PMC3051327

[10] Aziz RK Bartels D Best AA DeJongh M Disz T Edwards RA Formsma K Gerdes S Glass EM Kubal M Meyer F Olsen GJ Olson R Osterman AL Overbeek RA McNeil LK Paarmann D Paczian T Parrello B Pusch GD Reich C Stevens R Vassieva O Vonstein V Wilke A Zagnitko O 2008 The RAST server: Rapid Annotations using Subsystems Technology. BMC Genomics 9:75. 10.1186/1471-2164-9-75 18261238 PMC2265698

[11] Baba T Takeuchi F Kuroda M Yuzawa H Aoki K Oguchi A Nagai Y Iwama N Asano K Naimi T Kuroda H Cui L Yamamoto K Hiramatsu K 2002 Genome and virulence determinants of high virulence community-acquired MRSA. Lancet 359:1819–1827. 10.1016/S0140-6736(02)08713-5 12044378

[12] Zhou Y Liang Y Lynch KH Dennis JJ Wishart DS 2011 PHAST: a fast phage search tool. Nucleic Acids Res. 39:W347–W352. 10.1093/nar/gkr485 21672955 PMC3125810

[13] Bae T Baba T Hiramatsu K Schneewind O 2006 Prophages of *Staphylococcus aureus* Newman and their contribution to virulence. Mol. Microbiol. 62:1035–1047. 10.1111/j.1365-2958.2006.05441.x 17078814

[14] Baba T Bae T Schneewind O Takeuchi F Hiramatsu K 2008 Genome sequence of *Staphylococcus aureus* strain Newman and comparative analysis of staphylococcal genomes: polymorphism and evolution of two major pathogenicity islands. J. Bacteriol. 190:300–310. 10.1128/JB.01000-07 17951380 PMC2223734

[15] Li M Du X Villaruz AE Diep BA Wang D Song Y Tian Y Hu J Yu F Lu Y Otto M 2012 MRSA epidemic linked to a quickly spreading colonization and virulence determinant. Nat. Med. 18:816–819. 10.1038/nm.2692 22522561 PMC3378817

